# Feasibility of using self-reported patient data in a national diabetes register

**DOI:** 10.1186/s12913-015-1226-0

**Published:** 2015-12-15

**Authors:** Karianne Fjeld Løvaas, John G. Cooper, Sverre Sandberg, Thomas Røraas, Geir Thue

**Affiliations:** Norwegian Diabetes register for adults, Norwegian Center for Quality Improvement of Primary Care Laboratories (NOKLUS), Boks 6165, 5892 Bergen, Norway; Department of Global Public Health and Primary Care, University of Bergen, Postboks 7804, 5018 Bergen, Norway; Laboratory of Clinical Biochemistry, Haukeland University Hospital, Postboks 1400, 5021 Bergen, Norway; Haraldsplass Deaconess Hospital, Postboks 6165, Postterminal, 5892 Bergen, Norway

**Keywords:** Diabetes, Registers, Self-report, Questionnaires, Diabetes complications

## Abstract

**Background:**

In order to improve recruitment of patients to the Norwegian diabetes register for adults, a questionnaire was designed to collect data directly from patients. The main aim of this study was to assess the agreement of questionnaire data with data reported to the Register from health care personnel during routine consultations.

**Methods:**

Patient data were obtained by sending a questionnaire with 27 of the 41 Register variables to 3714 members of the Norwegian Diabetes Association. Questionnaire data were compared with data already in the Register. Paired t-tests, percentages of total agreement, percentages of “positive” answers and kappa coefficients (k) were used for comparing data.

**Results:**

Of the 1645 replies (44.3 %), the Register already had data on 324 patients for comparison. Response rate for most variables was better from patients (ranging from 76–100 %) compared with health care professionals (33–100 %). For 17 of 25 assessable variables including diabetes duration, height, weight, HbA1c, drug treatment and several diabetes complications, agreement was substantial or better with kappa >0.60. Data on family history of premature heart disease (k–0.59), foot examination (k = 0.26), foot ulcer (k = 0.32) and arterial surgery (k = 0.24) seemed to be difficult to answer by patients, whereas data on physical activity and self-monitoring of glucose seemed to be better when reported by patients.

**Conclusions:**

Patient response rate was acceptable, and data had good concordance with data from health care professionals for most variables. However, registers using patient questionnaires should compare questionnaire data with data from professionals at regular intervals.

## Background

The prevalence of diabetes has increased rapidly in Norway as well as worldwide [1, 2]. In Norway approximately 4 % [3] of the population have diabetes, and the majority (85–90 %) of these have type 2 diabetes. Diabetes management is complex and studies have revealed that a substantial portion of the patients not treated according to guidelines [4, 5]. The Norwegian diabetes register for adults (18 years or older) was established in 2006 with the purpose of improving diabetes care, reducing diabetes complications, and enabling comparisons of several clinical outcome measures between treatment centres. The Norwegian Government funds the Register. General practitioners, hospital outpatient clinics and diabetologists practicing outside hospitals are encouraged to report data collected electronically during consultations to the Register, but participation is voluntary. Patients have to give written consent before their data can be included in the Register.

In 2010 the Register was in an early phase of development and had data on only 4 % of the known diabetes population in Norway (approximately 11 % of the type 1 diabetes population and 3 % of the type 2 diabetes population). We therefore wanted to explore the feasibility of using a questionnaire to collect information directly from patients and thereby increase recruitment to the Register. We are not aware that any other national diabetes register has used this recruitment method. It is important that the quality of data in a register is high and there is a need for continuous validation [6].

Several studies have assessed the agreement between self-reported data and medical records [7–9], however only a few studies have included diabetic patients, and none has used register data for comparison. Register data collected during consultations may be more “interactive” than regular medical record data. Further, most studies contain only a few variables such as diabetes duration, use of insulin, other antidiabetic therapy or antihypertensive therapy, HbA1c and eye-examination [7–9]. Some studies are based on patient interviews rather than questionnaires [8, 9]. The main aim of this study was to assess the agreement of a comprehensive set of patient questionnaire data with data collected electronically for the diabetes register during routine consultations.

## Methods

### Data collection

Hospital outpatient clinics and almost all General Practitioners use electronic medical records. This allows the Norwegian diabetes register for adults to capture data electronically using a specially designed diabetes medical record and data capture application at outpatient clinics, and a pop-up window (a less complicated diabetes record and data capture system) at the General Practitioners’ offices. Data is registered during regular follow-up consultations by doctors or nurses, and is transferred electronically to the Register in January every year for the preceding year.

Patient data for comparison was obtained by distributing a questionnaire and one reminder to people with diabetes who were members of the Norwegian Diabetes Association in three counties in northern Norway. The Norwegian Diabetes association is a voluntary, independent patient organization. The Association provides information, advice and support to help people manage their diabetes as well as campaigning to improve the quality of diabetes care and supporting research. The members are people with diabetes and their family members, health care workers and others with a special interest in diabetes. The counties were selected because they already had a relatively high percentage of patients enrolled in the Register (10 % of their estimated diabetes population, compared to 4 % for the whole country) thus making comparisons between register data and self-reported data possible.

The questionnaire data were obtained between August 2010 and March 2011. Register data obtained from health care professionals were mostly from 2010, however data on diabetes duration, type of diabetes, height, family history of diabetes, completed educational course for type 2-diabetes, and ethnicity could be from both 2009 and 2010. A complete list of variables and definitions in the diabetes register can be viewed on the Noklus (Norwegian Quality Improvement of Primary Health Care Laboratories) home site [10], the organization responsible for the day-to-day operation of the Register.

### Variables

The questionnaire included 27 of the 41 variables in the Register. Variables that were considered to be difficult for the patient to understand or remember, such as cholesterol-values and the presence of foot pulses, were excluded. The wording of some of the register questions was modified to make them more readily understood. In this process we were guided by phrases used in a Norwegian health study (HUNT) [11]. The questionnaire was piloted on patients attending one general practice, and some minor changes were made. An overview of variables in the questionnaire is shown in Table 1.

**Table 1:** Overview of variables in the questionnaire

Basic data	Diabetes duration (years), height, weight, HbA1c, type of diabetes (type I/II), family history of diabetes, family history of premature coronary heart disease, participation in educational course for type 2 diabetes, ethnicity (white/African/Asian/other)
Health habits	Smoking habits (current smoker, ex-smoker for more than three months, never smoked), physical activity (at least 30 minutes of brisk walking or similar activity once a week ), self-monitoring of blood glucose at least once a week
Eye- and foot examination	Date of examination by an eye specialist (month/year), date of foot examination (not further specified) by a doctor (month/year)
Treatment	Insulin including insulin device, other antidiabetic therapy, antithrombotic therapy, lipid lowering therapy, antihypertensive therapy
Complications	Coronary heart disease, laser treated retinopathy, stroke, foot ulcer, arterial lower limb surgery, severe hypoglycemia (i.e. in need of assistance), amputation

### Statistics

We decided to compare data without using health care professionals’ data as the gold standard as there was some time-lag between collection of patients’ and health care professionals’ data. In addition some information, e.g. data on health habits, might be more reliable when obtained directly from patients. If the Register had data from more than one source, the most recent information was used in the comparison. Eye- and foot examination data were excluded if reporting dates indicated that results were unavailable to either patients or health care professionals and thus making comparison impossible. No data had to be excluded for other variables because of a time discrepancy.

A paired t-test with a level of significance of <0.05 was used to compare continuous data such as mean diabetes duration, height, weight and HbA1c. The percentages of agreement, percentages of “positive answers” and the kappa coefficient were used as measures of concordance for categorical data. Binominal confidence intervals were used to compare percentages of positive answers between the two groups such as percentages of eye- and foot-examinations. Data for all variables with differences between patients and health care professionals were divided by type of diabetes (type 1 and type 2, respectively) to see if differences were related to type of diabetes. The kappa coefficient was interpreted as follows: <0.21 was considered poor to slight agreement, 0.21–0.40 fair agreement, 0.41–0.60 moderate agreement, 0.61–0.80 substantial agreement, and 0.81–1.00 almost perfect agreement [12]. The Kappa coefficient corrects for chance agreement, but is sensitive to extremes in prevalence [13].

### Ethical approval

The Register is approved by the Norwegian Data Inspectorate. The Regional Committee for Medical and Health Research Ethics decided that approval for the project was unnecessary as the study was a quality assurance project.

## Results

A total of 3714 members with diabetes of the Diabetes Association in the three counties received the questionnaire, and 1645 replied (44.3 %). There was no difference in age, sex or type of diabetes between responders and non-responders based on membership information. We found that the Register had data on 324 of these patients (178 women and 146 men) for comparison with the self-reported data. One hundred and eighty-one had register data only from hospital outpatient clinics, 128 only from general practices, and 15 had data from both general practices and clinics. Overall, 39 % had type 1 diabetes, and 61 % had type 2 diabetes. As expected when the majority of register data comes from hospital clinics, there were more patients with type 1 diabetes among the 324 patients in the comparison group than among the responders to the questionnaire. Mean age for type 1 and type 2 diabetes were 49.1 and 64.1 years respectively. For Register data from health care professionals, the response rate varied from 33 % for questions regarding self-monitoring of blood glucose to 100 % on insulin administration. The response rate from the patients varied from 76 % on questions concerning HbA1c-results to 100 % on insulin administration. For almost all variables the response rate was substantially higher from patients compared with information obtained from health care professionals in the Register.

### Basic data

Tables 2 and 3 depict the agreement for continuous data, i.e. diabetes duration, height, weight and HbA1c. Self-reported data showed significantly longer mean diabetes duration (16.9 vs. 16.1 y) and lower mean weight (82.8 vs. 84.2 kg) compared with register data. The mean diabetes duration stated by patients was significantly longer compared to mean diabetes duration stated by health care professionals only for type 2 diabetic patients (11.5 y vs. 10.1 y) (data not shown).

**Table 2:** Comparison (health care professional vs. patients) of data on diabetes duration, height, weight and HbA1c

Basic data, part I	Diabetes duration (year)	Height (cm)	Weight (kg)	HbA1c (%)
Reported from professionals (mean/median)	16.1/12	171.2/171	84.2/84	7.5/7.3
Self-reported (mean/median)	16.9/14	171.3/171	82.8/82	7.5/7.2
Difference in mean	-0.78a	-0.09	1.42a	0.0

a) *p* < 0.05

**Table 3:** Percentage of agreement for diabetes duration, height, weight and HbA1c

Differences in duration (years), height (cm), and weight (kg)^a^	Percentage agreement related to the categories stated for				Differences in HbA1c –values (%)^a^
	Duration (*n* = 292)	Height (*n* = 269)	Weight (*n* = 263)	HbA1c (*n* = 240)	
≥5 (y/cm/kg)	1.1	1.9	11.4	5.4	≥1
>2,<5	2.4	2.2	17.9	5.8	>0.5, <1
≥-2, ≤2	85.6	91.4	66.5	80.4	≥-0.5, ≤0.5
>-5,<-2	4.5	3.3	3.4	5.0	>-1, <-0.5
≤ -5 (y/cm/kg)	6.5	1.1	0.8	3.3	≤ -1
Total (%)	100	100	100	100	

^a^Health care professional - minus self-reported

There was no difference in mean HbA1c, but for 8 % of the patients the HbA1c difference was 1.0 % or larger (Table 3). Agreement was not related to type of diabetes. Fourteen percent of patients with type 1 diabetes and 31 % of patients with type 2 diabetes were unaware of their last HbA1c value.

The agreement was substantial (k = 0.65) with respect to educational courses for type 2 diabetes with a match (portion of agreement) for 98/119 patients (Table 4). Regarding type of diabetes there was a mismatch for nine patients, but the files indicated probable faulty reporting not only from patients but also from health care professionals. Agreement on family history was almost perfect for diabetes (k = 0.82), but moderate for premature heart disease (k = 0.59) (Table 4). Ethnicity could not be assessed as almost no patients were in the “non-white” category.

**Table 4:** Comparison (health care professional vs. patients) of some basic data, and data on health habits, examinations, drug use and complications

	N	Response rate; HCP vs. P (%)^a^	Positive answers; HCP vs. P (%)^a^	Percentage of agreement	Kappa	(95 % CI)
Basic data (part II)						
Type of diabetes	312	99 vs. 97	-	97.1	0.94	(0.90, 0.98)
Family history of diabetes	250	80 vs. 95	51 vs. 50	91.2	0.82	(0.75, 0.89)
Attended educational course for type 2 diabetes	119	66 vs. 98	48 vs. 47	82.4	0.65	(0.51, 0.78)
Family history of premature heart disease	249	82 vs. 93	29 vs. 22	84.3	0.59	(0.48, 0.71)
Health habits						
Smoking habits	209	71 vs. 95	20 vs. 20	91.4	0.86	(0.81, 0.92)
Physical activity	168	57 vs. 99	80 vs. 84	83.3	0.43	(0.26, 0.61)
Self-monitoring of blood glucose	93	33 vs. 99	96 vs. 94	95.7	0.58	(0.20, 0.95)
Eye- and foot examination						
Eye-examination	153	66 vs. 91	71 vs. 79	84.3	0.58	(0.43,0.73)
Foot-examination	171	68 vs. 82	95 vs. 83	84.8	0.26	(0.03,0.48)
Drug use						
Insulin	312	98 vs. 97	67 vs. 68	96.8	0.93	(0.88, 0.97)
Pump/pen	206	100 vs. 100	-	99.5	0.99	(0.96, 1.00)
Other antidiabetic therapy	299	95 vs. 97	43 vs. 44	95.0	0.90	(0.85, 0.95)
Antithrombotic therapy	225	64 vs. 96	47 vs. 45	94.7	0.89	(0.83, 0.95)
Lipid lowering therapy	227	63 vs. 97	63 vs. 67	92.5	0.84	(0.76, 0.91)
Antihypertensive therapy	231	63 vs. 99	62 vs. 60	91.8	0.83	(0.75, 0.90)
Complications						
Coronary heart disease	294	92 vs. 99	16 vs. 19	93.5	0.78	(0.68, 0.87)
Hypoglycaemia (severe)	109	34 vs. 99	13 vs. 15	94.5	0.77	(0.59,0.95)
Laser-treated retinopathy	282	89 vs. 98	11 vs. 15	94.3	0.75	(0.63, 0.86)
Stroke	290	91 vs. 98	3 vs. 3	98.3	0.70	(0.44, 0.95)
Foot ulcer	231	63 vs. 97	3 vs. 7	93.5	0.32	(0.06, 0.59)
Arterial surgery	243	66 vs. 98	3 vs. 3	95.5	0.24	(-0.06, 0.54)

N: datasets

^a^Health care professionals vs. patients

### Health habits

The response rate from health care professionals compared with patients was lower for smoking habits and physical activity, and strikingly lower for self-monitoring of blood glucose (33 vs. 99 %). Agreement was almost perfect for smoking habits (k = 0.86), but moderate for physical activity (k = 0.43) and self-monitoring of blood glucose (k = 0.58) (Table 4).

### Eye and foot-examination

One hundred and fifty-three of the 324 patients (47.2 %) in the study had comparable data on eye examinations from both datasets, and agreement was moderate (k = 0.58). The patients did not report significantly more examinations than health care professionals.

The agreement on foot-examination was fair (k = 0.26) (Table 4). A significantly higher percentage of foot-examinations were reported from health care professionals than from patients.

### Treatment

Drug treatment generally showed a high level of agreement with kappa-values ranging from 0.83 to 0.93 (Table 4). In the 13 instances where only patients reported using lipid-lowering therapy, 11 had stated the name of the statin used. There was no difference in time-lag in registration between patients who had a match for lipid-lowering therapy and patients with a mismatch, and agreement regarding statin use was better for people with type 1 diabetes (k = 0.97, 95 % CI: 0.91, 1.00) compared to people with type 2 diabetes (k = 0.72, 95 % CI: 0.60, 0.85).

### Complications

The agreement was substantial for coronary heart disease (k = 0.78), severe hypoglycemia (k = 0.77), laser-treated retinopathy (k = 0.75) and stroke (k = 0.70), but only fair for foot ulcer (k = 0.32) and arterial lower limb surgery (k = 0.24) (Table 4). There were 17 foot ulcers reported from patients and only 6 from health care professionals. The patients reported only 2 of the 8 arterial interventions reported by health care professionals, whereas health care professionals reported only 2 of 7 interventions stated by patients. Amputation was not assessed due to very few events (data not shown).

## Discussion

### Main findings

The results indicate that for the majority of variables there was good concordance between registry data and self-reported data. All the continuous variables (Table 2) and most categorical variables showed substantial to almost perfect agreement with k > 0.60 (Table 4). For family history of premature heart disease, physical activity, self-monitoring of blood glucose and eye-examination agreement was moderate. For foot examination, foot ulcer and arterial surgery agreement was fair. For almost all questions the response rate was better from the patients. The percentage of patients completing questionnaires was acceptable.

### Comparison with other studies and explanation of the results

#### Basic data

The finding that type 2 diabetic patients tend to report longer mean diabetes duration is similar to the studies of Midthjell [7] and Wada [14], although these studies did not relate findings to type of diabetes. The explanation may be that the onset of type 1 diabetes is more acute and dramatic compared with type 2 diabetes and therefore more easily remembered. Self-reported weight was lower, and this has also been reported in another study that compared self-reported and actual measurements [15]. However, data in the Register may be a mixture of weight reported to health care professionals and actual measurements, and some of the differences may be explained by home measurements without shoes and clothes.

Although HbA1c (response rate 76 %) was the variable with the lowest response rate in the patient questionnaire, other studies have shown that only 24–48 % had knowledge of their HbA1c value [9, 16]. Our study also showed good concordance for HbA1c-values in contrast to a previous study with primary care patients [9]. Only 8 % had an absolute difference of ≥1 percentage points (Table 3) which exceeds the difference of 0.5–1.0 % found to be clinically important in a study assessing critical differences between HbA1c results [17]. Patients answering the questionnaire in our study were members of the Norwegian Diabetes Association and this may explain the high percentage who were aware of their last HbA1c level.

The findings on family history are similar to other studies with a substantial to almost perfect agreement on diabetes [18] but moderate agreement on premature heart disease [19]. Two more studies on family history of heart disease have shown slight to fair agreement [18, 20], suggesting that this is a difficult question for the patient to answer, and that discussion with a health care professional may be needed to get a valid answer.

#### Health habits

We found almost perfect agreement for smoking habits in line with a comparable study by Tisnado [21]. A Canadian study on 4530 patients that compared self-reported smoking habits with urine samples to document smoking [22] also reported good agreement.

For physical activity and self-monitoring of blood glucose the agreement was moderate, but might be more valid when reported on a questionnaire than directly to a health care professional.

#### Eye- and foot examination

Our study showed moderate agreement for eye examination, but could not replicate the findings by Fowles [8] and Beckles [23] that patients tend to report (significantly) more eye examinations than health care personnel because records tend to miss information provided outside the clinic. However, few datasets were available for comparison in our study.

The fair agreement for foot-examination in a study by Tisnado [21] is confirmed in our study. Our study showed a higher percentage of foot-examinations compared to other studies [24, 25], possibly since only “yes” or “no” responses could be included, excluding missing data (no response) which may indicate that the examination had not been performed. The response rate among the patients was relatively low, suggesting that the date (month/year) of the last foot-examination is difficult to remember.

#### Drug treatment

Our study showed almost perfect agreement for all medications, in line with other studies [7, 8, 21]. In instances of mismatch, patient data were probably correct since a correct drug name was stated. Although we checked for the influence of time lag, reporting date differences may still account for some of the mismatches, or an alternative explanation could be that the drug file in the electronic medical records had not been updated. A further investigation comparing the Register data with data in the Norwegian Prescription Database is planned.

#### Complications

Our study showed substantial agreement for coronary heart disease, severe hypoglycemia, laser treated retinopathy and stroke, and fair agreement (with large confidence intervals) for foot ulcer and arterial surgery. We have categorized myocardial infarction, angina and bypass/blocking into a yes/no for coronary heart disease whilst other studies have assessed how patients remember each of these coronary artery disease complications. Previous studies have shown an agreement on myocardial infarction from moderate to substantial [19, 21, 26–28] and slight to moderate for angina [19, 29]. Regarding stroke, previous studies have shown a great variation and differ from fair to almost perfect agreement [19, 26, 28, 29].

Our study had better agreement for laser treated retinopathy compared with previous studies that compared all types of retinopathy, probably since laser treatment was easier to remember [21, 30]. For severe hypoglycemia we are not aware of similar studies. Arterial surgery is not a well-known complication to patients, and the fair agreement may indicate that this is a difficult question for the patient to answer. Our study confirmed the fair agreement on foot ulcer reported by Tisnado due to more ulcers being reported by patients [21]. Patients may also report smaller ulcers, which may be unrelated to diabetes, or not reported to health care personnel.

### Limitations and strengths of the study

Patient data were obtained from members of the Norwegian Diabetes Association who may have had better knowledge of their disease than the general diabetes population, and possibly more frequent follow-up by health care professionals as many had type 1-diabetes. Further, a discrepancy in reporting dates between self-reported data and data collected from health care professionals may have occasionally led to mismatches.

On the other hand, the study obtained a high response rate from the patients for the majority of variables in the Register, and we were able to examine agreement between self-reported data and data from health care professionals for several variables that have not been evaluated in previous studies (type of diabetes, self-monitoring of blood glucose, hypoglycemia and participation in an educational course). We also explored differences in reporting related to type of diabetes for all variables. Only variables with differences (diabetes duration and lipid lowering therapy) are further discussed in the article.

## Conclusions

Our study obtained an acceptable response rate to a questionnaire with a comprehensive set of variables about diabetes care. There was substantial or near perfect agreement (kappa > 0.60) between self-reported data and data reported to the register by health care professionals for 17 of 25 assessable variables. We suggest that registers can consider using self-reported data to obtain these variables. Variables with moderate or poor concordance (kappa < 0.60) such as family history of premature heart disease, foot examination, foot ulcer and arterial surgery should be obtained from health care workers. Self-reported data on physical activity and self-monitoring of blood glucose might be more reliable than data reported to the register by health care workers. Registers using patient questionnaires should compare questionnaire data with data from professionals at regular intervals to ensure data quality.
